# Time Course of Cerebrovascular Reactivity in Patients Treated for Unruptured Intracranial Aneurysms: A One-Year Transcranial Doppler and Acetazolamide Follow-Up Study

**DOI:** 10.1155/2018/6489276

**Published:** 2018-04-26

**Authors:** Marianne Lundervik Bøthun, Øystein Ariansen Haaland, Nicola Logallo, Frode Svendsen, Lars Thomassen, Christian A. Helland

**Affiliations:** ^1^Department of Clinical Medicine, University of Bergen, 5020 Bergen, Norway; ^2^Department of Neurosurgery, Haukeland University Hospital, 5021 Bergen, Norway; ^3^Department of Global Public Health and Primary Health Care, University of Bergen, 5020 Bergen, Norway; ^4^Department of Neurology, Haukeland University Hospital, 5021 Bergen, Norway

## Abstract

**Background:**

Cerebrovascular reactivity (CVR) is often impaired in the early phase after aneurysmal subarachnoid hemorrhage. There is, however, little knowledge about the time course of CVR in patients treated for unruptured intracranial aneurysms (UIA).

**Methods:**

CVR, assessed by transcranial Doppler and acetazolamide test, was examined within the first postoperative week after treatment for UIA and reexamined one year later.

**Results:**

Of 37 patients initially assessed, 34 were reexamined after one year. Bilaterally, baseline and acetazolamide-induced blood flow velocities were higher in the postoperative week compared with one year later (*p* < 0.001). CVR on the ipsilateral side of treatment was lower in the initial examination compared with follow-up (58.9% versus 66.1%, *p* = 0.04). There was no difference in CVR over time on the contralateral side (63.4% versus 65.0%, *p* = 0.65). When mean values of right and left sides were considered there was no difference in CVR between exams. Larger aneurysm size was associated with increased change in CVR (*p* = 0.04), and treatment with clipping was associated with 13.8%-point increased change in CVR compared with coiling (*p* = 0.03).

**Conclusion:**

Patients with UIA may have a temporary reduction in CVR on the ipsilateral side after aneurysm treatment. The change in CVR appears more pronounced for larger-sized aneurysms and in patients treated with clipping. We recommend that ipsilateral and contralateral CVR should be assessed separately, as mean values can conceal side-differences.

## 1. Introduction

Constriction and dilation of cerebral arterioles regulate cerebral blood flow. Cerebrovascular reactivity (CVR) reflects this regulating capacity and is a marker of cerebrovascular integrity. Impaired CVR is associated with increased risk of cerebro- and cardiovascular disease and death [1]. The temporal development of CVR has been studied in healthy subjects and in patients with cerebrovascular disease. In healthy persons, CVR is stable over time [2]. In the early phase after aneurysmal subarachnoid hemorrhage (aSAH), CVR is often impaired [3–8], especially in patients with massive hemorrhage, poor neurological status at admission, and vasospasm [9–15]. It has been suggested that transient reduction of CVR after aSAH may be associated with development of delayed cerebral ischemia and poor outcome [9, 16, 17]. There is, however, little knowledge regarding the time course of CVR in patients with unruptured intracranial aneurysms (UIA). It is unknown whether, and how, aneurysm treatment affects CVR. Information on the time course of CVR in patients treated for UIA may help in differentiating between potential effects of aneurysm treatment and the impact of an aneurysm bleeding.

The main objective of this study was to evaluate the time course of CVR in patients treated for an UIA by comparing CVR within the first week after aneurysm treatment with CVR one year later. We further wanted to assess whether other factors like age, sex, smoking, hypertension, body mass index, aneurysm size, treatment side, or treatment modality were associated with the stability of CVR over time.

## 2. Methods

### 2.1. Participants and Time Scheme

In a previous study, we analyzed early postoperative CVR data from patients treated for UIA in the Department of Neurosurgery, Haukeland University Hospital, between February 2011 and May 2013 [18]. The patients were treated with either endovascular coiling or surgical clipping, and they were examined within the first week after aneurysm treatment. In the present study, CVR was reevaluated in the same patients one year after aneurysm treatment. Exclusion criteria were identical to those used in the previous study: former treatment of intracranial aneurysms; nonsaccular aneurysms; giant aneurysms treated with proximal artery occlusion; carotid stenosis (>50%) or occlusion; lack of transtemporal bone window in transcranial Doppler examination; and contraindications to acetazolamide (e.g., sulfonamide allergy, adrenal or pituitary insufficiency, and kidney or liver failure).

Demographics, aneurysm location, and treatment were recorded, as well as body mass index, smoking status, and hypertension (previously diagnosed and treated or systolic pressure > 140 mmHg and/or diastolic pressure > 90 mmHg persistently observed during admission). Aneurysm size was measured using the following parameters: maximum diameter of the dome, independent of angles and directions (maximum diameter,* D*max), maximum diameter of the dome, perpendicular to the aneurysm height (width, *W*), maximum height from dome tip perpendicular to aneurysm neck (height, *H*), and diameter of the aneurysm neck (neck, *N*). Aspect ratio (*H*/*N*) and bottleneck ratio (*W*/*N*) were calculated [19, 20].

The study was conducted in accordance with the Declaration of Helsinki (2013) of the World Medical Association and was approved by the local ethics committee. All patients gave written informed consent.

### 2.2. Cerebrovascular Reactivity

CVR testing was performed using transcranial Doppler (TCD) monitoring of blood flow velocities in the middle cerebral arteries (MCA) before, during, and after intravenous injection of acetazolamide (AZ). The method has previously been described in detail [18]. Except for an additional manufacturer of AZ, the method of CVR testing was identical to the initial study [18]. The AZ manufacturers used in this study were Goldshield Ltd., Croydon, Surrey, UK; Sanofi Aventis, Paris, France; and Mercury Pharmaceuticals Ltd., Croydon, Surrey, UK. The AZ dose was 1000 mg for patients weighing < 80 kg, and 15 mg/kg for patients weighing ≥ 80 kg. The maximum dose was 1500 mg. All examinations in the initial and follow-up study were performed by the same sonographer (MLB).

Cerebrovascular reactivity was defined as the maximum percentage change in mean blood flow velocity (MFV) after administration of AZ: CVR  (%) = [(MFV_AZ_ − MFV_BASELINE_)/MFV_BASELINE_] × 100, where CVR is cerebrovascular reactivity, MFV_BASELINE_ is baseline mean blood flow velocity (before AZ), and MFV_AZ_ is maximum mean blood flow velocity after AZ.

### 2.3. Statistical Analysis

Two measures of central tendency and dispersion were used: mean and standard deviation (SD) for variables that were symmetric around the mean, and median and interquartile range (IQR) for those that were nonsymmetric. In cases where patients underwent treatment for multiple aneurysms during the same procedure, averaged aneurysm size was used in the analyses. The relationships between blood flow velocities and CVR at the time of initial examination and one year later were studied using paired *t*-tests. Paired *t*-test was also used to assess possible differences in velocities and CVR related to side (right/left and ipsilateral/contralateral to the aneurysm treatment). Regarding treatment modality (clipping or coiling), two-sample *t*-tests were used. To simplify analyses, patients with midline aneurysms were allocated to the side chosen for endovascular or surgical approach. As in the previous study [18], mean CVR of the two sides (right and left) was calculated for all individuals. If the measurement on one side was missing, the mean CVR was set to the nonmissing value. Simple linear regressions, stratified on treatment modality, were conducted on mean CVR at follow-up versus mean CVR at first examination. Also, difference in mean CVR between first exam and follow-up was the outcome in a multiple regression and a set of simple linear regressions. Covariates were age, sex, hypertension, smoking, body mass index, weight difference from initial exam to follow-up, treatment modality, maximum aneurysm diameter (*D*max), mean CVR at the time of initial examination, and difference in mean AZ dose per kg from initial exam to follow-up. Lastly, maximum aneurysm diameter was included as covariate in a simple linear and multiple regression with CVR at the time of the initial examination as outcome, in addition to the covariates tested in a previous report (age, sex, hypertension, smoking, body mass index, and treatment modality) [18]. The regression analyses were repeated with ipsilateral CVR as outcome variable instead of mean CVR, and with stratification for age, sex, and treatment modality.

All statistical analyses were performed with R version 3.4.3 [21].

## 3. Results

### 3.1. Patients, Aneurysm, and Treatment

Of 37 patients examined in the initial study, two patients chose to abstain from the follow-up test due to side effects of AZ at the initial examination, and one patient did not meet for follow-up due to long travel to the hospital. This left us with a study population of 34 patients.

Table 1 shows patient characteristics. Weight difference is the difference in body weight from the first examination to follow-up. All other variables listed in the table were recorded at the time of aneurysm treatment. Mean age was 49.0 (SD 9.6, range 27–65) years. In 20 of the 34 patients (58.8%) the body weight was different at the time of follow-up compared with the first examination (range 10 kg reduction–13 kg increase). In 11 patients (32.4%) the weight difference was >2 kg.  Table 2 shows aneurysm and treatment characteristics.

### 3.2. Cerebrovascular Reactivity

In total, 56 bilateral and 12 unilateral examinations were performed in the 34 patients. Unilateral examinations were more common in the initial exams (23.5%) compared with follow-up (11.8%), presumably because postoperative intracranial air can cause insufficient insonation. Median time between treatment and initial exam was 51.0 (IQR 39.5) hours. Median time between treatment and follow-up exam was 376.5 (IQR 31.8) days. Median time between initial examination and follow-up was 374.5 (IQR 29.3) days.

Of 68 examinations, 42 (61.8%) were performed using 1000 mg AZ. The remaining 26 examinations (38.2%) were done with 15 mg AZ per kg because of high bodyweight. Mean bodyweight was 76.1 (SD 15.5, range 40 to 110) kg at the time of the first examination and 76.6 (SD 17.2, range 40 to 118) kg at follow-up. Mean AZ dose was 15.1 (SD 2.3) mg/kg in the first examination and 15.1 (SD 2.4) mg/kg at follow-up. There was no correlation between AZ dose per kg and CVR (Pearson's *R* = 0.08, *p* = 0.66 in the first examination, and *R* = −0.11, *p* = 0.52 at follow-up).

Table 3 shows blood flow velocities and CVR results. In the initial examination MFV in the middle cerebral arteries was 58.6 cm/s before stimulation with AZ and 94.3 cm/s after, giving a mean CVR of 62.7%. Follow-up testing showed MFV 51.4 cm/s before AZ, 84.4 cm/s after, and mean CVR of 65.6%. Bilaterally, baseline and AZ-induced blood flow velocities were higher in the postoperative week compared with 1 year after aneurysm treatment (*p* ≤ 0.009 in all situations). When assessing mean values of the right and left sides, no difference between CVR at first examination and follow-up was found (*p* = 0.31). When assessing CVR according to treatment laterality, there was no difference over time on the contralateral side (65.0% at follow-up versus 63.4% at the initial examination, *p* = 0.65). However, on the ipsilateral side of aneurysm treatment there was an apparent change in CVR over time. Ipsilateral CVR was 58.9% (SD 19.3) in the initial examination versus 66.1% (SD 18.5) at follow-up (*p* = 0.04), corresponding to an absolute increase of 7.2% and relative increase of 12%. Subgroup analyses for treatment modalities had lower sample sizes, and the significance disappeared (*p* = 0.16). CVR change on the ipsilateral side seemed larger in patients treated with clipping compared with patients treated with coiling (absolute increase 10.5 versus 4.7%, and relative increase 17 versus 8%), yet the number of patients in each subgroup is low (*n* = 12 for clipping and *n* = 22 for coiling) and results are inconclusive (*p* = 0.42). The same trend was found for mean CVR values of the right and left side, with an absolute and relative increase of 8.7% and 13% in patients treated with clipping versus 0.2% absolute reduction and 0% relative change in patients treated with coiling (*p* = 0.18). The tendency of larger CVR difference between exams was present for all patients treated with aneurysm clipping, regardless of whether temporal clipping of a parent artery was performed or not. However, despite similar values for CVR difference between exams, patients treated with temporal clipping appeared to have higher CVR values compared with patients treated with “standard” clipping (without the need for temporary clipping), at both the initial exam and follow-up. Due to few observations, statistical power is however insufficient to evaluate potential differences within the clipping subgroup.

Table 4 shows the results of the regression analyses regarding the relationship between difference in mean CVR from the first examination to follow-up and several different variables. In the simple analysis, maximum aneurysm diameter and CVR in the first examination were associated with a change in CVR. An 1 mm increase in the maximum diameter of the aneurysm dome was associated with an increase in CVR difference by 3.2 and 2.5 percentage points in the simple and multiple regressions, respectively (*p* = 0.005 and *p* = 0.04). For every percentage point increase in CVR in the first examination, the change in CVR from initial exam to follow-up was reduced with 0.3 percentage points (*p* = 0.05). The association was stronger in the multiple model, where the reduction in CVR change was 0.5 percentage points for every percentage point increase in CVR in the first examination (*p* = 0.01). In the multiple analyses age and treatment modality were also associated with change in CVR. For age, the change in CVR increased with 0.8 percentage points per year (*p* = 0.03). For treatment modality, the multiple model showed that patients treated with clipping had 13.8 percentage points increased change in CVR compared with patients treated with coiling (*p* = 0.03). There were no associations between change in CVR and sex, body mass index, body weight difference between exams, hypertension, and smoking. There were no major changes in the results when regression analyses were repeated after stratification for sex, age (≤50 years versus >50 years), and treatment modality (coiling versus clipping).

Regression analyses were also repeated with ipsilateral CVR as outcome variable instead of mean CVR. Patients with missing CVR values on the ipsilateral side, at the time of either the first exam or follow-up, were excluded. This applied to 7 of 34 (20.6%) patients: 2 of 22 (9.1%) patients treated with coiling and 5 of 12 (41.7%) patients treated with clipping. The lower sample size in the analyses with ipsilateral CVR as outcome yielded more uncertainty. Apart from higher *p* values, findings were primarily consistent with the results of the original regressions using mean CVR as outcome. Maximum aneurysm diameter and CVR at first exam were still associated with change in ipsilateral CVR between exams in the simple analysis (*p* = 0.05), whereas the multiple analysis provided weaker evidence for such associations (*p* = 0.22 for aneurysm diameter and *p* = 0.10 for first CVR). The positive association between change in CVR and age and clipping found in the regression with mean CVR was less obvious in the regression with ipsilateral CVR. The estimate for age was 0.7 (*p* = 0.10) versus 0.8 (*p* = 0.03) in the regression with mean values. The estimate for clipping was 9.8 (*p* = 0.19) in the regression with ipsilateral values versus 13.8 (*p* = 0.03) in the regression with mean values.

Finally, regression analyses were performed to assess if larger aneurysm diameter was correlated with lower initial CVR. When only results from the initial exam were included in the statistical analyses, fewer observations yielded high *p* values. Maximum aneurysm diameter had an estimate of −0.6 in the simple analysis (*p* = 0.61) and −1.7 in the multiple analysis (*p* = 0.15). Few observations hamper the assessment of a possible association between larger aneurysm size and reduced CVR in the first week after aneurysm treatment.

**Figure 1:**
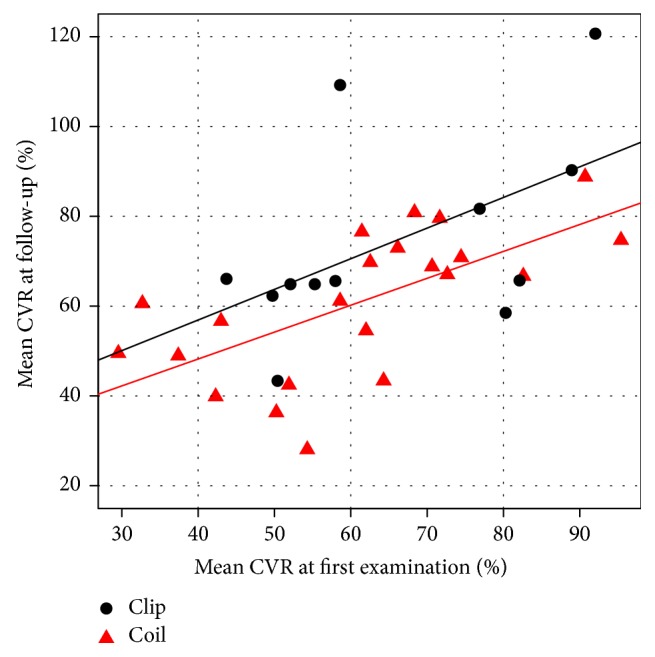
Scatter plot of mean cerebrovascular reactivity (CVR) at the time of initial examination and follow-up, together with regression lines. Results for patients treated with coiling are marked with red triangles, and results for patients treated with clipping are marked with black dots.

Figure 1 shows a scatter plot of mean CVR at first exam and follow-up. The regression lines for the two treatment modalities are almost parallel, with the line for patients treated with clipping shifting up about 8 to 15 percentage points. This is in accordance with Table 4.

**Figure 2:**
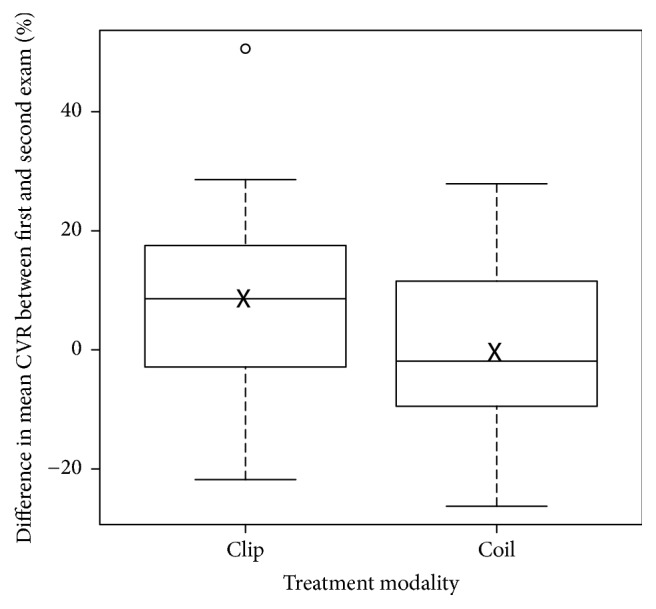
Box plots comparing change in mean cerebrovascular reactivity (CVR) from the first examination to follow-up in patients treated with coiling and clipping. Boxes extend from the 25th to the 75th percentile. Horizontal bars represent the median, and whiskers extend to the most extreme point that is less than 1.5 times the interquartile range from the box. Mean values are marked with crosses, and a single outlier is depicted as a circle.

Figure 2 shows box plots comparing change in mean CVR from first exam to follow-up in patients treated with coiling and clipping. Although there was little evidence for difference in mean CVR change in patients treated with coiling compared with clipping (*p* = 0.14), Figure 2 hints that patients treated with clipping had a greater difference between the initial examination and follow-up. Still, the evidence is inconclusive.

Diamox Goldshield Ltd. was used in 44 CVR tests (64.7%), Sanofi Aventis in 20 tests (29.4%), and Mercury Pharmaceuticals Ltd. in 4 tests (5.9%). There did not seem to be any important differences in CVR between the three manufacturers (Mercury versus Goldshield: *p* = 0.07; Mercury versus Sanofi Aventis: *p* = 0.84; Goldshield versus Sanofi Aventis: *p* = 0.46).

## 4. Discussion

### 4.1. Main Findings

In this study, we found a lower CVR on the ipsilateral side of aneurysm treatment in the postoperative week compared with one-year follow-up. There was no evidence of any difference in CVR over time when mean values of the right and left sides were assessed. Larger aneurysm size is associated with increased change in CVR. In addition, results suggest that the difference in CVR may be greater in patients treated with clipping compared with coiling, but evidence is inconclusive.

In a previous study, where CVR was examined in the first week after aneurysm treatment, we did not find any difference when comparing treated and untreated sides (59.4% versus 63.0%, *p* = 0.16) [18]. We concluded that CVR in patients with UIA did not differ from normal values reported in healthy subjects and that findings did not indicate a systemically impaired vascular system in patients with UIA. New information based on results from follow-up testing one year later now indicates that there may be a side difference in CVR after aneurysm treatment after all. Ipsilateral CVR was 58.9% after treatment versus 66.1% one year later, and contralateral CVR was 63.4% after treatment versus 65.0% one year later. The postoperative CVR of 58.9% seems to stand out as lower than the other CVR values, indicating a temporary reduction in CVR on the treated side. Even though we could not rule out the fact that the trend with lower CVR on the ipsilateral side was due to chance when only postoperative results were assessed (*p* = 0.16) the difference was more pronounced when follow-up results were included (difference in ipsilateral CVR over time, *p* = 0.04). Still, the sample size is limited and results must be interpreted with caution.

Furthermore, this study showed higher baseline and AZ-induced blood flow velocities in the postoperative week compared with one year after aneurysm treatment. The increased velocities can be explained by postoperative hyperemia. Transient hyperemia is common after craniotomy [22]. However, hyperemia has previously only been found in the first postoperative hour [22], and the median time for the first CVR testing in our study was 51.0 (IQR 39.5) hours after treatment. To our knowledge, there have been no reports about transient hyperemia after endovascular aneurysm treatment. Alternatively, posttreatment spasm could be the cause of elevated blood flow velocities at the initial exam. Moreover, comparison of absolute blood flow velocities is problematic as the probe positioning and insonation angle probably were different at the time of the initial and follow-up exam. Still, there is no reason why altered insonation angle and probe positioning should only cause increased velocities. In theory, changes in technical insonation aspects could just as well cause a reduction of measured velocities.

Our finding of reduced CVR on the ipsilateral side after aneurysm treatment may be related to postoperative hyperemia. It is possible that a transient hyperemia in response to aneurysm clipping or coiling affects the arteriolar vasodilating capacity and thus influences CVR. Baseline velocities (MFV_BASELINE_) were higher at first exam compared with one-year follow-up (59.8 cm/s versus 51.2 cm/s, *p* < 0.001), whereas the absolute change in velocity (ΔMFV) was the same at the two examination times (33.8 cm/s versus 33.1 cm/s, *p* = 0.43). Since CVR is defined as percentage change in velocity after AZ compared with baseline, the same ΔMFV yields lower CVR values when baseline velocities are increased (unchanged numerator and higher denominator of the fraction). An alternative explanation for decreased CVR after aneurysm treatment may be that harboring an aneurysm in itself impairs CVR. This effect may still be present in the first postoperative days, while vasodilating capacity can be restored after aneurysm treatment and CVR normalized one-year later. Since TCD and AZ testing were not performed prior to aneurysm treatment it is difficult to say if the reduction in CVR is caused by the aneurysm itself or by aneurysm treatment.

### 4.2. Time Course of CVR in Healthy Subjects

Schwertfeger et al. (2006) assessed the time course of CVR in healthy subjects [2]. TCD and AZ were used to investigate CVR in 33 healthy subjects at baseline and after 1 to 3 years (mean 21.6 months). They performed unilateral testing and found no changes in CVR over time. Like in our study, they did not find any association between sex and smoking and CVR change. Unlike their findings, we found a positive association between age and change in CVR from first examination to follow-up (*p* = 0.03 in the multiple regression model). The possible influence by age on CVR is unclear, and studies in healthy subjects have shown varying effects [23–30].

### 4.3. Time Course of CVR in Patients with Intracranial Aneurysms

Several studies have assessed the time course of CVR in patients with ruptured intracranial aneurysms. CVR is often impaired in the early phase after aSAH [3–6], especially in patients with massive hemorrhage, poor neurological status at admission, and vasospasm [9–15]. There is a possible association between progressive impairment in CVR in the early phase after aSAH and subsequent development of delayed cerebral ischemia [9, 16]. Transient reduction of CVR may also be associated with poor outcome [17]. Follow-up studies months and years after aSAH have shown normalization of CVR, regardless of the severity of hemorrhage and presence of vasospasm in the acute phase [31–33]. In contrast to the numerous studies on CVR in patients with aSAH there are few studies addressing CVR in patients with UIA. To our knowledge only one study has used AZ test and TCD to assess CVR in this patient group [18], and six reports have used CO_2_ as vasoactive stimuli [3, 4, 7, 14, 15, 33]. In these studies, CVR testing was performed either at a single time-point after aneurysm treatment [18, 33] or at multiple time-points within 24 hours in close relation to the time of aneurysm surgery [3, 4, 7, 14, 15]. To our knowledge, this is the first study to investigate within-subject differences over time in patients treated for UIA. We found evidence in favor of a transient reduction of CVR on the ipsilateral side of aneurysm treatment, and recommend separate assessment of ipsilateral and contralateral CVR as mean values of right and left sides can conceal side-differences. Our finding of possible transient reduction of CVR also after treatment for* unruptured* aneurysms provides valuable insight and may enable better interpretation of CVR results after aneurysm treatment.

Studies with measurement of CVR at a single time-point have not shown any association between CVR and aneurysm treatment modality [18, 31], whereas in this follow-up study a possible association between treatment modality and change in CVR was found. Patients treated with aneurysm clipping appeared to have a larger difference in CVR between exams. This tendency was present for all patients treated with aneurysm clipping, regardless of whether temporal clipping of a parent artery was performed or not. Still, the number of patients in the subgroups for treatment modality is low. In particular, the number of observations in patients treated with clipping is reduced due to missing values, presumably related to postoperatively intracranial air. Results should thus be interpreted with caution.

### 4.4. Technical Considerations

Blood flow velocities demonstrate diurnal variations [34]. The follow-up examination was not performed at the identical time of day as the initial CVR test, but the time differences between exams were small (median 2.25 hours, IQR 2.56 hours) and we consider their influence as negligible. Mean AZ dose was the same in the first examination and follow-up (mean 15.1 mg/kg, SD 2.3 and 2.4 mg/kg, respectively). A third of the patients had a weight difference of >2 kg from first exam to follow-up. Nonetheless, the weight difference was small (mean 0.4 kg, SD 4.7 kg) and there was no association between weight difference and change in CVR (*p* = 0.93). We used different brands of AZ, but there was no differences in CVR between the three manufacturers. As expected, insufficient insonation due to postoperative intracranial air was more common on the ipsilateral side of aneurysm clipping compared with the contralateral side, or compared with patients treated with coiling.

### 4.5. Strengths and Limitations

To our knowledge, this is the first study to investigate within-subject differences in CVR over time in patients treated for UIA. The sample size is in the upper range compared with CVR studies in healthy subjects [2, 35–46]. The method of testing was identical at initial examination and follow-up. We used the same sonographer (MLB) in all examinations to reduce operator variability, as the intrarater reproducibility for TCD examinations has been found superior to interrater [47, 48].

The AZ dose should ideally have been bodyweight-based in all patients in the study, not only in patients weighing ≥80 kg. Still, the recommended AZ dose of 13 to 18 mg/kg [39, 49] was achieved for the vast majority of patients (91.2%). Blood flow velocities are affected by physiological factors such as hematocrit, arterial CO_2_ tension, heart rate, and mean arterial pressure [50], and hyperventilation can theoretically counteract the vasodilatory effect of AZ. We did not routinely monitor these parameters in our study.

Even though the sample size is rather large compared with other CVR studies, the limited number of patients makes it difficult to draw definite conclusions regarding regression results and subgroup effects, especially for treatment modality. The regression analyses based on ipsilateral CVR were hampered by missing values in several patients, especially in patients treated with clipping. Subgroup analysis based on laterality and treatment modality should be considered when planning the sample size of future studies.

To best assess the effect of aneurysm treatment on CVR it would have been preferable to examine patients before and after the procedure. Patients were not examined before aneurysm treatment in our study, partly because this study was part of a larger study where the set-up was designed for comparison of CVR in patients treated for ruptured and unruptured aneurysms, and partly because we wanted to avoid test-induced aneurysm rupture, a highly unlikely yet serious complication.

## 5. Conclusions

This study implies that patients with UIA may have a temporary reduction in CVR on the ipsilateral side after aneurysm treatment. The change in CVR is associated with larger aneurysm size and is possibly more pronounced in patients treated with clipping. We recommend that results from ipsilateral and contralateral sides should be assessed separately as mean values can conceal side-differences in CVR.

## Figures and Tables

**Table 1:** Patients characteristics.

	(*n* = 34)
Age, years^a^	49.0 (9.6)
Height, cm^a^	169.2 (8.6)
Weight, kg^a^	76.1 (15.5)
BMI, kg/m^2^^a^	26.5 (4.8)
Weight difference, kg^a^	0.4 (4.7)
Female^b^	22 (64.7)
Hypertension^b^	15 (44.1)
Smoking^b^	
Current	19 (55.9)
Previous	11 (32.4)
Never	4 (11.8)

^a^mean (SD); ^b^*n* (%); BMI: body mass index; SD: standard deviation.

**Table 2:** Aneurysm and treatment characteristics.

	*n* = 34
Multiple aneurysms, *n* (%)	12 (35.3)
Treatment modality, *n* (%)	
Coil	22 (64.7)
Clip^a^	12 (35.3)
Treatment side, *n* (%)^b^	
Left	18 (52.9)
Right	16 (47.1)
Location of treated aneurysms, *n* (%)	
MCA	14 (41.2)
ICA, incl. ophthalmic artery and PCOM	10 (29.4)
ACOM, anterior complex, and pericallosal artery	7 (20.6)
Basilar top, cerebelli superior, PICA, VB, and distal posterior	3 (8.8)
Size of treated aneurysms, mean (SD)^c^	
Maximum diameter (*D*max), mm	6.3 (2.4)
Height (*H*), mm	6.4 (2.8)
Neck (*N*), mm	4.1 (1.8)
Width (*W*), mm	5.4 (2.2)
Aspect ratio (*H*/*N*)	1.6 (0.5)
Bottleneck ratio (*W*/*N*)	1.4 (0.5)

^a^In four of twelve patients temporal clipping of a parent artery was performed; ^b^one patient treated with combined clipping of an ACOM aneurysm and a right MCA aneurysm in one procedure was allocated to the right side. Eight patients with midline aneurysms (ACOM and basilar top) were allocated to the chosen side of approach; ^c^the majority of patients received treatment for a single aneurysm. For the 4 of 34 patients (11.8%) that underwent treatment for two aneurysms during the same procedure, aneurysm size was averaged; ACOM: anterior communicating artery; ICA: internal carotid artery; MCA: middle cerebral artery; PCOM: posterior communicating artery; PICA: posterior inferior cerebellar artery; VB: vertebrobasilar artery; maximum diameter (*D*max): maximum diameter of the dome (independent of angles and directions); height (*H*): maximum height from dome tip perpendicular to aneurysm neck; neck (*N*): diameter of the aneurysm neck; width (*W*): maximum diameter of the dome, perpendicular to the aneurysm height (*H*).

**Table 3:** Blood flow velocities and cerebrovascular reactivity at baseline and follow-up.

	First exam	Follow-up	Absolute difference	Relative difference	*p*
	mean (SD)	mean (SD)			
MFV_BASELINE_ (cm/s)					
Ipsilateral	59.8 (13.8)	51.2 (13.3)	−8.6	0.86	<0.001
Contralateral	57.7 (14.7)	52.0 (12.1)	−5.7	0.90	0.003
Mean	58.6 (13.2)	51.4 (11.7)	−7.2	0.88	<0.001
MFV_AZ_ (cm/s)					
Ipsilateral	93.6 (18.0)	84.4 (20.2)	−9.2	0.90	0.001
Contralateral	93.4 (23.3)	85.0 (20.1)	−8.4	0.91	0.009
Mean	94.3 (20.0)	84.4 (18.5)	−9.9	0.90	<0.001
ΔMFV (cm/s)					
Ipsilateral	33.8 (9.2)	33.1 (9.8)	−0.7	0.98	0.43
Contralateral	35.7 (11.2)	33.0 (11.8)	−2.7	0.93	0.15
Mean	35.7 (10.2)	33.1 (9.9)	−2.7	0.93	0.09
CVR (%)					
Ipsilateral	58.9 (19.3)	66.1 (18.5)	7.2	1.12	0.04
Contralateral	63.4 (17.5)	65.0 (23.6)	1.7	1.03	0.65
Mean	62.7 (17.2)	65.6 (19.4)	2.9	1.05	0.31
MV (*n*)					
Ipsilateral	7	3			
Contralateral	1	1			

CVR: cerebrovascular reactivity; MFV_BASELINE_: baseline mean blood flow velocity (before acetazolamide); MFV_AZ_: maximum mean blood flow velocity after acetazolamide; ΔMFV: absolute change in mean flow velocity after acetazolamide; MV: missing value; *p*: *p* value from paired *t*-test (follow-up–first); SD: standard deviation. Note that if one side had a missing value, the mean is just the remaining value. This is why the mean is not simply the mean of ipsilateral and contralateral values. Patients with MV were excluded from the paired *t*-test. The sample size was therefore reduced in the analyses of ipsilateral and contralateral values (ipsilateral *n* = 27, contralateral *n* = 33, mean *n* = 34).

**Table 4:** Regression results: difference in mean CVR between exams versus a number of variables.

	Simple		Multiple	
	Estimate	*p*	Estimate	*p*
Age, years	0.1	0.68	0.8	0.03
Female	−4.9	0.42	−4.5	0.46
BMI, kg/m^2^	−0.2	0.78	−0.1	0.84
Weight difference, kg	0.3	0.63	0.3	0.81
Hypertension	1.7	0.78	3.3	0.58
Smoking				
Current	ref	-	ref	-
Previous	−2.8	0.67	−2.8	0.62
Never	−5.2	0.59	−0.7	0.94
Treatment modality				
Coil	ref	-	ref	-
Clip	9.0	0.14	13.8	0.03
Maximum aneurysm diameter (*D*max)	3.2	0.005	2.5	0.04
Mean CVR at first exam, %	−0.3	0.04	−0.5	0.01
Difference in AZ dose per kg	−2.4	0.49	−1.9	0.81

AZ: acetazolamide; BMI: body mass index; CVR: cerebrovascular reactivity; *D*max: maximum diameter of the aneurysm dome (independent of angles and directions); ref: reference.
